# Trends and Predictors of Premature Termination of Cardiovascular Trials: A Systematic Review

**DOI:** 10.1016/j.jacadv.2026.102606

**Published:** 2026-03-16

**Authors:** Frederick Berro Rivera, Nathan Ross B. Bantayan, John Vincent Magalong, Chieh-Mei Tsai, Nicole Tesoro, Polyn Luz S. Pine, Neill Steven Cainglet Cachuela, Sung Whoy Cha, Christine J. Lin, Vuyisile T. Nkomo, Mandeep Singh, Naveen L. Pereira, Kyla Lara-Breitinger, Mohamad Alkhouli, Mayra Guerrero

**Affiliations:** a Division of Cardiovascular Medicine, Mayo Clinic, Rochester, Minnesota, USA; b University of the Philippines College of Medicine, Manila, Philippines; c College of Medicine, San Beda University, Manila, Philippines; d Department of Medicine, NYC Health + Hospitals/South Brooklyn Health, New York, New York, USA; e Ateneo School of Medicine and Public Health, Manila, Philippines; f Faculty of Medicine and Surgery, University of Santo Tomas, Manila, Philippines; g Cebu Institute of Medicine, Cebu City, Philippines; h Rush Medical College, Chicago, Illinois, USA

**Keywords:** cardiovascular diseases, clinical trials, ClinicalTrials.gov, early termination, patient recruitment

## Abstract

**Background:**

Premature termination of cardiovascular trials undermines evidence generation and wastes resources.

**Objectives:**

The objective of the study was to evaluate trends, predictors, and recruitment adequacy among prematurely terminated cardiovascular trials.

**Methods:**

We analyzed adult cardiovascular trials registered on ClinicalTrials.gov (2000-2025). Trial characteristics, early termination, and reported reasons were extracted. Computed low recruitment was defined as actual enrollment <80% of target. Multivariable logistic regression identified independent predictors of termination.

**Results:**

Among 19,191 trials, 2,202 (11.5%) were prematurely terminated. Low recruitment was the most common reported reason (946/2,202, 42.9%), yet 625/1,571 (39.9%) underenrolled trials did not report recruitment failure. Termination peaked in 2020 (155/1,033, 15.0%). Termination risk was the highest in early-phase trials (phases 1/2 or 2; 371/2,325, 16.0%) and lowest for behavioral/lifestyle interventions (146/2,998, 4.9%) and female-only trials (23/475, 4.8%). In multivariable analysis, small sample size (1-100 participants; OR: 3.37; 95% CI: 2.98-3.80) and later-phase trials (phase 2/3 or 3; OR: 1.69; 95% CI: 1.41-1.97) were associated with higher odds of termination, whereas behavioral/lifestyle interventions (OR: 0.40; 95% CI: 0.32-0.49), crossover designs (OR: 0.53; 95% CI: 0.42-0.65), and non-U.S. government funding (OR: 0.50; 95% CI: 0.31-0.78) were protective.

**Conclusions:**

More than 1 in 10 cardiovascular trials terminate early, most often due to poor recruitment, which is frequently under-reported. Improved feasibility assessment and transparent reporting are needed. (Trends and Predictors of Premature Termination of Cardiovascular Trials: A Systematic Review; CRD420251155096)

Randomized controlled trials underpin evidence-based cardiovascular medicine but are often discontinued before completion, leaving them underpowered and wasting scientific and financial resources.^1^^,^^2^ Recruitment shortfalls have long been recognized as a central cause of trial failure.^3^, ^4^, ^5^, ^6^, ^7^ Early evaluations of publicly funded multicenter studies in the United Kingdom found that only 31% of trials achieved their target enrollment between 1994 and 2002, and nearly half required extensions in the subsequent decade, confirming the persistence of enrollment struggles.^3^^,^^4^ Similar findings emerged from the Netherlands, where 17.8% of drug trials registered from 2007 to 2015 were discontinued, with recruitment failure (5.7%), futility (5.4%), and efficacy (5.2%) being the most common reasons.^5^ For cardiovascular trials specifically, prior analyses of ClinicalTrials.gov (2000-2015) reported termination rates of 10% to 15%, with low recruitment accounting for 40% to 55% of discontinued studies and a higher risk among academically funded trials.^6^^,^^7^ Recent analyses of 51 major cardiovascular outcome trials found that although U.S. sites represented one-quarter of global trial locations, they achieved the lowest patient accrual rate of median 0.2 patients per site per month.^8^ These findings highlight inefficiencies in global recruitment strategies, particularly among U.S. sites, where legal and logistical barriers contribute to persistently low patient accrual.^8^

Barriers to recruitment are multifactorial, spanning both patient-level and trial-level factors.^9^ Among these, several patient-factors are modifiable including lack of interest and poor compliance.^10^ Consistently, there are multiple preventable trial-level factors that affect participation, such as overly complex protocols, and inadequate recruiter engagement.^11^ However, prior investigations show that many terminated trials fail to report recruitment outcomes or justify early closure, limiting the understanding of systemic barriers.^12^ Sex-specific analyses and reporting remain limited in cardiovascular trials, despite well-documented sex differences in treatment response, adverse drug reactions, and premature study drug discontinuation, potentially influencing recruitment and retention.^13^^,^^14^

No study has comprehensively examined trends in cardiovascular trial termination across the modern era of trial registration. We therefore analyzed cardiovascular trials registered on ClinicalTrials.gov from 2000 to 2025 to characterize temporal trends, predictors, and discrepancies between reported and objectively measured recruitment failure, providing an updated framework to inform future trial design and transparency efforts.

## Methods

We searched the ClinicalTrials.gov database to identify cardiovascular clinical studies registered from January 1, 2000, to March 31, 2025. Clinical studies were defined as interventional trials in which participants are assigned to ≥1 intervention to evaluate health outcomes.^15^ Eligible interventions included drug, device, biologic, behavioral, and lifestyle interventions, as defined by ClinicalTrials.gov. Cardiovascular diseases were defined using Medical Subject Headings (MeSH) and included coronary artery disease, cardiomyopathies, heart valve diseases, pericardial diseases, arrhythmias, pulmonary heart disease, hypertension, stroke, and other vascular conditions.^16^ Only adult populations (≥18 years) were included.

Initial screening for cardiovascular relevance was assisted by a large language model (GPT-4.1-mini, 2023, OpenAI) applied to trial titles and condition fields using a prespecified MeSH-aligned prompt (full prompts and scripts in Supplemental Appendix A and B). Trials were classified as include, exclude, or manual review. Model outputs were constrained to structured JSON format and generated using deterministic settings (temperature = 0). Trials flagged for manual review, missing key fields, or returning invalid outputs were adjudicated by human reviewers. Trials were classified into completed or terminated per ClinicalTrials.gov status. For terminated trials, investigator-reported reasons for termination were retrieved through the ClinicalTrials.gov definitions. For terminated trials, declared reasons for termination were retrieved through the ClinicalTrials.gov application programming interface. Free-text reasons were categorized into prespecified groups using an artificial intelligence (AI)-assisted workflow with deterministic settings; outputs indicating multiple reasons or ambiguous classification were flagged for manual adjudication (taxonomy and prompts in Supplemental Appendix A and B). Low recruitment was additionally defined objectively as actual enrollment <80% of anticipated enrollment.^3^ For trials without reported termination reasons, PubMed searches using National Clinical Trial numbers and linked publications were performed to verify trial status and determine discontinuation causes when available.

Extracted trial characteristics included sponsor type, intervention class, sample size, trial phase, design, masking, endpoint type, and age/sex categories. The sample size was based on anticipated enrollment. Actual enrollment was used only to calculate recruitment adequacy.^3^ Funding source was inferred from lead sponsor and collaborator fields using an AI-assisted sponsor classification workflow with standardized rules and caching to ensure consistency (details in the Supplemental Methods and Supplemental Appendix A). These classifications reflect organizational involvement rather than verified financial contribution.

A random 2% sample underwent independent manual validation by 2 investigators, with >98% agreement. Discrepancies were resolved by consensus adjudication.

Descriptive statistics summarized trial characteristics by completion status. Group differences were assessed using the Pearson chi-square test. Multivariable logistic regression identified independent predictors of premature termination (covariates listed in Supplemental Methods). Analyses were conducted using Stata 14 MP (StataCorp LLC) and Microsoft Excel (Microsoft Corp). A Preferred Reporting Items for Systematic reviews and Meta-Analyses flow diagram is provided in the Supplemental Figure. This systematic review was prospectively registered in PROSPERO (CRD420251155096).

## Results

### Trial completion trends

**Figure 1:**
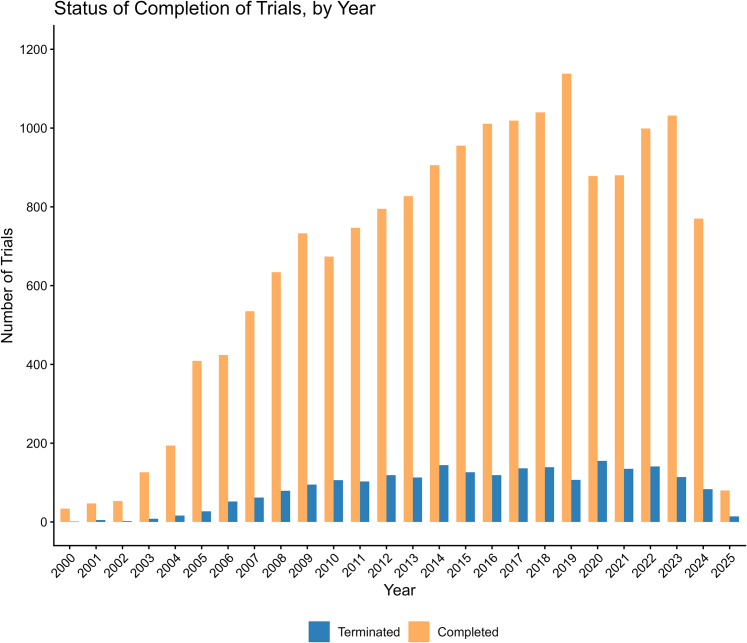
**Annual Completed vs Terminated Trials From 2000 to 2024** Annual distribution of cardiovascular trial registered on ClinicalTrials.gov (2000-2025) by completion status. Only trials categorized as completed or terminated were included; ongoing and withdrawn studies were excluded from this analysis. Year refers to the registration year of the study.

Among 19,191 cardiovascular trials identified between 2000 and 2025, 16,989 (16,989/19,191, 88.5%) were completed and 2,202 (2,202/19,191, 11.5%) were terminated early (Central Illustration). Trial registrations increased steadily through the 2000s, peaking in 2018, with a subsequent decline. Termination rates peaked at 15.0% (155/1,033) in 2020, coinciding with the onset of the COVID-19 pandemic, before partially recovering thereafter (Figure 1, Supplemental Table 1).

**Figure 2:**
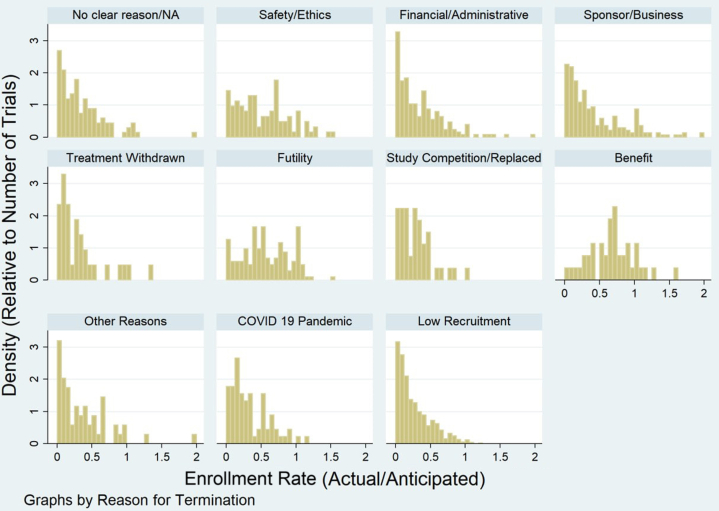
**Distribution of Actual vs Anticipated Enrollment Rates by Reported Reason for Termination** Histograms display the ratio of actual to anticipated enrollment across prematurely terminated cardiovascular trials, stratified by reason for termination. Each subplot represents a cited reason showing the relative density of trials with varying degrees of enrollment success.

**Central Illustration:**
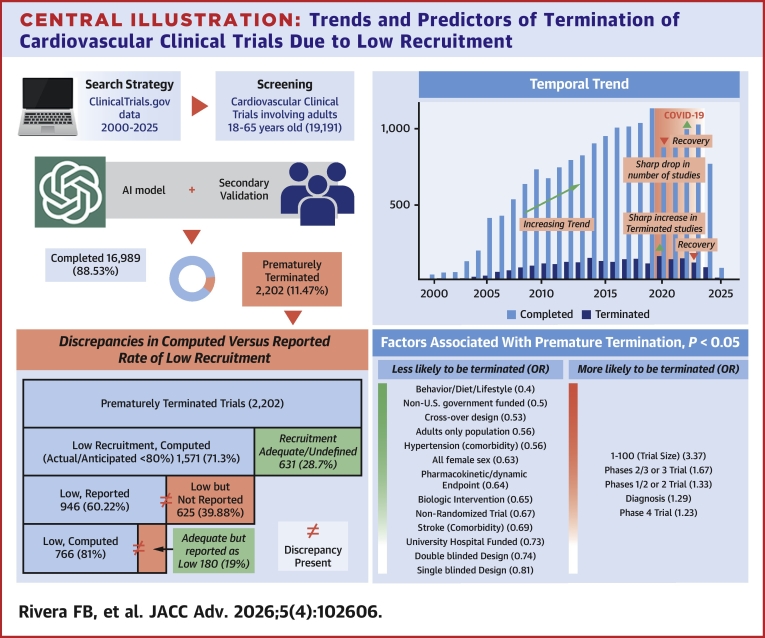
**Trends and Predictors of Termination of Cardiovascular Clinical Trials Due to Low Recruitment** Summary of 19,191 cardiovascular trials (2000-2025), illustrating the study workflow, temporal trends in completion and premature termination (including the COVID-19 peak), discrepancies between computed and reported low recruitment, and trial characteristics associated with termination risk. Year refers to the registration year of the study. AI = artificial intelligence.

### Associations between trial characteristics and early termination

Termination rates differed significantly across trial characteristics (Table 1). Mixed-age trials exhibited higher termination than adult-only trials (1,997/16,512, 12.1% vs 58/1,111, 5.2%; *P* < 0.001), and mixed-sex trials had higher termination than female-only trials (2,139/18,243, 11.7% vs 23/475, 4.8%; *P* < 0.001). Heart failure/cardiomyopathy had the highest termination rate (341/2,220, 15.4%), whereas hypertension (162/2,373, 6.8%) and stroke trials (252/2,447, 10.3%) had lower rates (overall *P* < 0.001). Drug and device trials were terminated more frequently than behavioral or lifestyle trials (993/6,973, 14.2% and 595/4,208, 14.1% vs 146/2,998, 4.9%; *P* < 0.001). Small-sized trials (1-100-participant) showed markedly higher termination compared with medium-sized trials (1,669/11,361, 14.7% vs 437/6,383, 6.9%; *P* < 0.001). Early-phase trials (phases 1/2 or 2) also had higher termination rates than trials without phase designation (371/2,325, 16.0% vs 975/10,367, 9.4%; *P* < 0.001). Industry-sponsored trials demonstrated higher termination than university or non-U.S. government–sponsored trials (698/5,080, 13.7% vs 782/7,702, 10.2% and 22/297, 7.4%; *P* < 0.001).

**Table 1:** Characteristics of Clinical Trials, by Trial Completion Status

	Terminated	Completed	Total	%Terminated	Statistical Test
Comorbidity					
Multiple (ref)	183	1,483	1,666	11.0%	Pearson chi-square (6) = 100.4988 Pr = 0.000
Hypertension	162	2,211	2,373	6.8%	
Heart failure/cardiomyopathy	341	1879	2,220	15.4%	
Coronary artery disease/acute coronary syndrome	328	2,742	3,070	10.7%	
Arrhythmias, incl atrial fibrillation	189	1,195	1,384	13.7%	
Stroke	252	2,195	2,447	10.3%	
Others	747	5,284	6,031	12.4%	
Randomization					
Randomized (ref)	1,593	12,301	13,894	11.5%	Pearson chi-square (2) = 0.8006 Pr = 0.670
Nonrandomized	599	4,585	5,184	11.6%	
Not specified	10	103	113	8.9%	
Endpoints					
Safety and efficacy (ref)	324	2,212	2,536	12.8%	Pearson chi-square (5) = 50.3936 Pr = 0.000
Efficacy only	1,421	10,380	11,801	12.0%	
Safety only	149	983	1,132	13.2%	
Pharmacokinetic/pharmacodynamic	174	1934	2,108	8.3%	
Others	119	1,240	1,359	8.8%	
Not stated	15	240	255	5.9%	
Study design					
Parallel (ref)	1,476	10,762	12,238	12.1%	Pearson chi-square (5) = 46.7900 Pr = 0.000
Crossover	105	1,414	1,519	6.9%	
Factorial	38	436	474	8.0%	
Single arm	558	4,078	4,636	12.0%	
Sequential	13	155	168	7.7%	
Not available	12	144	156	7.7%	
Blinding					
Open label (ref)	1,148	8,621	9,769	11.8%	Pearson chi-square (4) = 48.3367 Pr = 0.000
Single blind	319	3,113	3,432	9.3%	
Double blind	275	2,400	2,675	10.3%	
Triple/quadruple blind	454	2,475	2,929	15.5%	
Not available	6	110	116	5.2%	
Purpose					
Treatment (ref)	1,548	10,512	12,060	12.8%	Pearson chi-square (4) = 78.8761 Pr = 0.000
Diagnosis	158	1,099	1,257	12.6%	
Prevention	223	2,212	2,435	9.2%	
Other/unclassified	273	3,166	3,439	7.9%	
Intervention					
Drug (ref)	993	5,980	6,973	14.2%	Pearson chi-square (4) = 235.3152 Pr = 0.000
Device	595	3,613	4,208	14.1%	
Biologics	35	258	293	12.0%	
Behavior/diet/lifestyle	146	2,852	2,998	4.9%	
Others/unspecified	433	4,286	4,719	9.2%	
Trial size					
1-100	1,669	9,692	11,361	14.7%	Pearson chi-square (3) = 283.6695 Pr = 0.000
101-1,000 (ref)	437	5,946	6,383	6.9%	
>1,000	86	1,206	1,292	6.7%	
Not available	10	145	155	6.5%	
Participation by sex					
Both males and females (ref)	2,139	16,104	18,243	11.7%	Pearson chi-square (3) = 26.0094 Pr = 0.000
All females	23	452	475	4.8%	
All males	40	432	472	8.5%	
Not available	0	1	1	0.0%	
Participation by age group					
Adults, including senior citizens (ref)	1,997	14,515	16,512	12.1%	Pearson chi-square (3) = 55.9163 Pr = 0.000
Adults only	58	1,053	1,111	5.2%	
Senior citizens only	36	331	367	9.8%	
Others, including children	111	1,090	1,201	9.2%	
Sponsor					
Industry (ref)	698	4,382	5,080	13.7%	Pearson chi-square (6) = 47.8721 Pr = 0.000
NIH	35	345	380	9.2%	
University hospital	782	6,920	7,702	10.2%	
Mixed source funding	532	4,169	4,701	11.3%	
Others	61	405	466	13.1%	
Non-U.S. government	22	275	297	7.4%	
Research institutes/nonprofit organization	72	493	565	12.7%	
Phase of trial					
Not applicable (ref)	975	9,392	10,367	9.40%	Pearson chi-square (4) = 129.9169 Pr = 0.000
Phases 0 (early phase 1)/1	106	1,009	1,115	9.51%	
Phases 1/2 or 2	371	1954	2,325	15.96%	
Phases 2/3 or 3	396	2,279	2,675	14.80%	
Phase 4	354	2,355	2,709	13.07%	

Percentages in the table indicate the proportion of terminated trials within each subgroup. Statistical significance of associations was assessed using the Pearson chi-square test. Both chi-square values and corresponding *P* values are shown. Factors such as small trial size, industry sponsorship, earlier-phase studies, nonrandomized design, and certain endpoint types were significantly associated with higher termination rates (*P* < 0.05).

NIH = National Institutes of Health.

### Predictors of low recruitment as the reported reason for termination

In a separate multivariable model assessing predictors of citing low recruitment as the reported reason for early termination (Table 2), small trial size (1-100 participants) was strongly associated with reporting low recruitment (OR: 3.37; 95% CI: 2.98-3.80; *P* < 0.01). Later-phase trials (phase 2/3 or 3) were also more likely to cite recruitment failure (OR: 1.67; 95% CI: 1.41-1.97; *P* < 0.01).

**Table 2:** Multivariable Logistic Regression for Odds of Citing Low Recruitment as the Reported Reason for Premature Termination

	OR^a^ (95% CI)	*P* Value
Comorbidity		
Multiple (ref)	Ref	Ref
Hypertension	0.56 (0.44-0.70)	<0.01
Heart failure/cardiomyopathy	1.07 (0.87-1.31)	0.52
Coronary artery disease/Acute Coronary Syndrome	0.77 (0.63-0.94)	0.12
Arrhythmias, incl atrial fibrillation	1.01 (0.81-1.27)	0.90
Stroke	0.69 (0.55-0.85)	<0.01
Others	0.93 (0.78-1.12)	0.45
Randomization		
Randomized (ref)	Ref	Ref
Nonrandomized	0.67 (0.55-0.83)	<0.01
Endpoints		
Safety and efficacy (ref)	Ref	Ref
Efficacy only	1.10 (0.96-1.26)	0.18
Safety only	0.94 (0.76-1.17)	0.6
Pharmacokinetic/pharmacodynamic	0.64 (0.52-0.79)	<0.01
Others	0.90 (0.71-1.15)	0.40
Study design		
Parallel (ref)	Ref	Ref
Crossover	0.53 (0.42-0.65)	<0.01
Factorial	0.77 (0.55-1.09)	0.15
Single arm	0.93 (0.76-1.14)	0.47
Sequential	0.63 (0.35-1.14)	0.13
Blinding		
Open label (ref)	Ref	Ref
Single blind	0.81 (0.69-0.93)	<0.01
Double blind	0.74 (0.63-0.87)	<0.01
Triple/quadruple blind	0.89 (0.77-1.02)	0.10
Purpose		
Treatment (ref)	Ref	Ref
Diagnosis	1.29 (1.06-1.56)	0.01
Prevention	0.97 (0.83-1.14)	0.72
Other/unclassified	0.87 (0.75-1.01)	0.07
Intervention		
Drug (ref)	Ref	Ref
Device	1.02 (0.88-1.19)	0.81
Biologics	0.65 (0.45-0.94)	0.02
Behavior/diet/lifestyle	0.40 (0.32-0.49)	<0.01
Trial size		
1-100	3.37 (2.98-3.80)	<0.01
101-1,000 (ref)	Ref	Ref
>1,000	0.82 (0.64-1.05)	0.12
Participation by sex		
Both males and females (ref)	Ref	Ref
All females	0.63 (0.40-0.98)	0.04
All males	1.04 (0.73-1.47)	0.83
Participation by age group		
Adults, including senior citizens (ref)	Ref	Ref
Adults only	0.56 (0.42-0.76)	<0.01
Senior citizens only	0.94 (0.66-1.35)	0.75
Others, including children	0.77 (0.62-0.95)	0.01
Sponsor		
Industry (ref)	Ref	Ref
NIH	0.94 (0.63-1.39)	0.76
University hospital	0.73 (0.64-0.83)	<0.01
Mixed source funding	0.90 (0.78-1.03)	0.13
Others	0.89 (0.67-1.20)	0.46
Non-U.S. government	0.50 (0.31-0.78)	<0.01
Research institutes/nonprofit organization	0.92 (0.70-1.20)	0.52
Phase of trial		
Not applicable (ref)	Ref	Ref
Phases 0 (early phase 1)/1	0.90 (0.71-1.14)	0.39
Phases 1/2 or 2	1.33 (1.13-1.58)	<0.01
Phases 2/3 or 3	1.67 (1.41-1.97)	<0.01
Phase 4	1.23 (1.04-1.45)	0.01

Model estimates the likelihood of low recruitment being cited as the reason for early discontinuation across trial characteristics. ORs >1 indicate increased likelihood of citing recruitment failure (protective), whereas OR <1 indicate reduced likelihood (harmful).

Multivariable logistic regression was performed to estimate adjusted ORs with 95% CIs for predictors of early termination. Covariates included sponsor type, trial phase, sample size, intervention type, randomization, blinding, primary endpoint, study design, age group, sex, and clinical indication.

NIH = National Institutes of Health.

a Multivariable logistic regression models were adjusted for sponsor type, trial phase, sample size, intervention type, randomization, blinding, endpoint type, study design, age group, sex, and clinical indication.

Conversely, behavioral or lifestyle interventions (OR: 0.40; 95% CI: 0.32-0.49; *P* < 0.01), crossover designs (OR: 0.53; 95% CI: 0.42-0.65; *P* < 0.01), pharmacokinetic/pharmacodynamic studies (OR: 0.64; 95% CI: 0.52-0.79; *P* < 0.01), female-only trials (OR: 0.63; 95% CI: 0.40-0.98; *P* = 0.04), adult-only populations (OR: 0.56; 95% CI: 0.42-0.76; *P* < 0.01), university hospital sponsorship (OR: 0.73; 95% CI: 0.64-0.83; *P* < 0.01), and non-U.S. government sponsorship (OR: 0.50; 95% CI: 0.31-0.78; *P* < 0.01) were significantly less likely to report low recruitment.

### Recruitment adequacy and reported reasons

Among 2,202 prematurely discontinued trials, 1,571/2,202 (71.3%) enrolled fewer than 80% of their target population (Table 3). Low recruitment was the most frequently reported reason for termination (946/2,202, 43.0%), followed by sponsor or business decisions (262/2,202, 11.9%), financial or administrative reasons (228/2,202, 10.4%), and futility (186/2,202, 8.4%).

**Table 3:** Comparison of Reported Reasons for Termination and Computed Recruitment Adequacy Among Terminated Cardiovascular Trials

Reported Reason for Early Termination	Recruitment Status (Actual/Anticipated Enrollment), x100%				
	Low <80%	Adequate ≥80%	Missing	Total	% Low Recruitment
Low recruitment (ref)	**766**	**73**	**107**	**946**	**81.0%**
Safety/ethics	73	29	14	116	62.9%
Financial/administrative	164	37	27	228	71.9%
Sponsor/business	173	47	42	262	66.0%
Treatment withdrawn from market	28	6	5	39	71.8%
Futility	115	52	19	186	61.8%
Study competition/replaced	37	8	7	52	71.2%
Benefit	27	16	5	48	56.3%
COVID 19 pandemic	52	21	8	81	64.2%
Other specified reasons	44	11	8	63	69.8%
No clear reason for termination	92	15	74	181	50.8%
TOTAL	**1,571**	**301**	**330**	**2,202**	**71.3%**

Distribution of actual enrollment performance among terminated cardiovascular trials, categorized by the cited reason for termination. Recruitment status is grouped as low (<80% of anticipated enrollment), adequate (≥80%), or not stated. The proportion of trials with low enrollment is the highest among those explicitly citing recruitment failure (80.97%) but also notable in trials terminated for other reasons such as financial issues, sponsor decisions, and the COVID-19 pandemic.

Percentages of “low recruitment” are calculated using all terminated trials as the denominator (including those with missing enrollment data), to reflect their proportion within the total terminated cohort.

“Missing” indicates trials without sufficient termination reason reported in ClinicalTrials.gov.

Trials explicitly citing low recruitment demonstrated the highest proportion of objective underenrollment (766/946, 81.0%). However, substantial underenrollment was also observed among trials terminated for financial or administrative reasons (164/228, 71.9%), sponsor or business decisions (173/262, 66.0%), and COVID-19–related disruptions (52/81, 64.2%). In contrast, trials terminated for benefit or safety exhibited lower proportions of underenrollment (27/48, 56.3% and 73/116, 62.9%, respectively), consistent with termination following interim efficacy or safety assessments (Figure 2).

### Characteristics of trials by termination reason

Across trial subgroups, low recruitment remained the predominant reason for premature termination (Supplemental Table 2). Arrhythmia trials most frequently cited low recruitment (96/189, 50.8%), followed by coronary artery disease trials (157/328, 47.9%). Male-only trials showed the highest proportion terminated for recruitment failure (26/40, 65.0%), whereas female-only trials were least affected (10/23, 43.5%). Diagnostic trials most often cited low recruitment (87/158, 55.1%), as did university-sponsored trials (414/782, 52.9%) and non-U.S. government–funded studies (12/22, 54.5%). In contrast, larger phase 3 or 4 trials more frequently terminated for futility (eg, 122/524, 23.3%) or sponsor decisions (110/524, 20.9%), reflecting adaptive stopping based on interim analyses rather than recruitment failure.

## Discussion

In this registry-based analysis of cardiovascular trials registered on ClinicalTrials.gov between 2000 and 2025, most trials were completed; however, premature termination affected a substantial minority, reflecting persistent challenges in trial execution. Poor participant recruitment was the leading cause of premature termination, although it was frequently under-reported. The temporary increase in termination observed during the COVID-19 pandemic further underscored the vulnerability of conventional trial infrastructures to external disruption.

Recruitment shortfalls have been repeatedly identified as the leading cause of cardiovascular trial failure, with prior analyses reporting termination rates of 10 to 15% and regional variation.^6^^,^^7^ Importantly, recruitment failure was frequently not identified as the primary reason for termination despite objective evidence of insufficient enrollment. This discordance suggests that commonly reported administrative, financial, or sponsor-related reasons may often represent downstream consequences of poor accrual rather than independent causes.^17^ Structural limitations of trial registries—particularly the requirement to assign a single primary reason for termination—may further obscure overlapping contributors to trial failure, underscoring the need for more standardized and transparent reporting.^18^^,^^19^

Several trial-level characteristics were associated with vulnerability to recruitment-related termination. Diagnostic-purpose studies and trials with smaller planned sample sizes were particularly susceptible, consistent with greater logistical complexity and limited feasibility assessment before trial initiation.^6^^,^^7^^,^^9^^,^^17^^,^^20^, ^21^, ^22^, ^23^, ^24^ Phase-related findings were mixed across prior studies,^6^^,^^7^^,^^17^ but in our adjusted analyses, later-phase trials demonstrated higher odds of recruitment-related termination, suggesting that accrual challenges persist even in large-scale cardiovascular research.^8^ In contrast, trials evaluating biologic or behavioral interventions, those using crossover or blinded designs, and studies supported by academic or non–U.S. government sponsors were less prone to recruitment failure, potentially reflecting lower participant burden, stronger investigator engagement, and more pragmatic trial designs.

Recruitment feasibility also varied by population and disease context. Female-only trials were least affected, potentially reflecting targeted recruitment strategies and reduced study burden.^21^ However, this finding must be interpreted in the context of persistent under-representation of women and limited sex-stratified reporting in cardiovascular trials, despite known sex differences in adverse drug reactions and treatment discontinuation.^13^^,^^14^^,^^25^, ^26^, ^27^, ^28^, ^29^ Women continue to comprise only ∼40% of participants in contemporary cardiovascular trials, with notable gaps in coronary, heart failure, cardiometabolic, and valvular intervention studies.^25^, ^26^, ^27^, ^28^, ^29^ Trials enrolling older adults or patients with symptomatic cardiovascular conditions faced inherent barriers related to frailty, comorbidity, and competing clinical demands.^30^, ^31^, ^32^, ^33^, ^34^, ^35^, ^36^, ^37^, ^38^, ^39^ In contrast, preventive studies embedded within established care pathways may benefit from broader eligibility criteria and clearer perceived benefit.^36^, ^37^, ^38^, ^39^

The pandemic-related increase in trial discontinuation highlights the limitations of site-dependent research models during periods of health system stress.^40^, ^41^, ^42^ Although few trials explicitly attributed termination to COVID-19, the broader operational impact was evident, including restricted hospital access, redeployment of research staff, and suspension of nonessential activities.^41^^,^^42^ Subsequent stabilization of termination rates likely reflects increasing adaptation to remote follow-up and decentralized trial components, approaches that may enhance trial resilience in future settings.^43^, ^44^, ^45^

Collectively, these findings indicate that premature termination of cardiovascular trials is not an isolated occurrence but a predictable outcome associated with modifiable design, feasibility, and reporting factors. Greater emphasis on pretrial feasibility assessment, improved transparency in termination reporting, and wider implementation of pragmatic and participant-centered trial designs may reduce avoidable discontinuation and improve the efficiency and ethical conduct of cardiovascular clinical research.^20^^,^^46^

### Study Limitations

This study relied on ClinicalTrials.gov data, which may be incomplete, inconsistent, or nonstandardized. ClinicalTrials.gov is the largest public registry of clinical studies, with U.S. interventional trials mandated to register and report results under the Food and Drug Administration Amendments Act of 2007. Nevertheless, reporting completeness and accuracy remain variable, particularly for older or non-U.S. studies. Only registered trials were included, possibly under-representing non-U.S. or industry-funded trials. Actual enrollment may not always reflect active recruitment cessation (eg, trials stopped early for efficacy or harm). Some trials may have achieved scientific adequacy despite enrolling fewer participants (eg, higher-than-expected event rates or larger effect sizes), but verifying such context would require publication-level review and was beyond the scope of this registry-based analysis. Discrepancies between reported and observed enrollment further suggest reporting inaccuracies. Because low enrollment can occur secondary to nonrecruitment reasons (eg, funding withdrawal or strategic termination), some classifications overlap between recruitment-related and other termination causes is possible. Our disease taxonomy followed MeSH-based cardiovascular definitions; diabetes and metabolic trials not primarily labeled as cardiovascular were grouped under “other,” possibly underestimating cardiometabolic studies. The “older age” classification was data set-driven rather than standardized (eg, ≥65 years), which may limit comparability across studies. Timing of primary completion and termination was often incompletely reported, limiting assessment of how early trials were discontinued and potentially introducing selection bias toward older, completed trials. Publication status was unavailable in registry data; linking terminated trials to subsequent publications was beyond this study’s scope. Sex-specific recruitment, dropout, and termination patterns could not be assessed, as ClinicalTrials.gov does not provide sex-disaggregated enrollment, retention, or adverse event data.

## Conclusions

About 1 in 10 cardiovascular trials were terminated early, most often due to low recruitment. Termination rates remained stable over 2 decades, peaking briefly during the COVID-19 pandemic. Smaller, later-phase, and complex trials were more likely to discontinue for recruitment failure, whereas behavioral and academically sponsored studies were less affected. Enhancing feasibility, infrastructure, and transparency is essential to reduce preventable discontinuations and improve research efficiency.

Perspectives **COMPETENCY IN MEDICAL KNOWLEDGE:** Premature termination remains a persistent challenge in cardiovascular clinical research, with approximately 1 in 10 trials discontinued—most commonly due to low recruitment. Smaller, later-phase, and complex studies are particularly vulnerable, whereas behavioral and academically sponsored trials are less affected. Enhancing feasibility assessment, site infrastructure, and recruitment monitoring may help mitigate these preventable losses and improve research efficiency.

**TRANSLATIONAL OUTLOOK:** Efforts to reduce premature discontinuation should focus on integrating feasibility metrics at trial design, adopting adaptive and decentralized recruitment strategies, and improving transparency in trial reporting. Future research should explore predictive models for accrual success and evaluate pragmatic frameworks that optimize participant accessibility while maintaining scientific rigor.

## Funding support and author disclosures

The authors have reported that they have no relationships relevant to the contents of this paper to disclose.
