# Supplementary material for: Trends and Predictors of Premature Termination of Cardiovascular Trials: A Systematic Review
Source: JACC Adv. 2026 Mar 16;5(4):102606. doi: 10.1016/j.jacadv.2026.102606 (PMC13131426; doi:10.1016/j.jacadv.2026.102606)
Supplement: Supplemental data 1 [file mmc1.docx]

**SUPPLEMENTS**

**eMethods. Detailed Search Strategy and Classification Procedures**

**Search Strategy and Study Selection**

We searched ClinicalTrials.gov for cardiovascular interventional trials registered between January 1, 2000, and March 31, 2025. Cardiovascular diseases included structural, ischemic, valvular, arrhythmic, pericardial, pulmonary heart, and vascular disorders, including cerebrovascular disease. The study search was limited to adults aged ≥18 years.

**AI-Assisted Cardiovascular Relevance Screening**

Cardiovascular relevance screening was performed using the OpenAI application programming interface (API; OpenAI, San Francisco, CA; 2023) with the gpt-4.1-mini model and a custom Python pipeline. The model evaluated trial titles and condition fields against MeSH-aligned criteria and returned Include, Exclude, or Manual Review labels in structured JSON format. Deterministic settings (temperature=0) and automated retry logic were applied. Trials with malformed outputs, missing decisions, or ambiguity were automatically flagged for manual adjudication. Prompts, definitions, and scripts are provided in **Supplementary Appendices A and B**.

From 25,296 retrieved records, 19,443 were included, 5,725 excluded, and 128 adjudicated manually.

**Classification of Trial Status and Termination Reasons**

Trials were categorized as completed or terminated per registry status. Terminated trials’ “why stopped” fields were retrieved via the ClinicalTrials.gov API. Reasons were categorized into prespecified groups using an AI-assisted workflow: low recruitment; safety or ethics issues;, sponsor or business decisions; financial or administrative reasons; futility; treatment benefit; study competition or replacement; treatment withdrawal from the market; disruptions related to COVID-19; or other/multiple reasons requiring manual review.

The prompt required assignment to a single category. Records indicating multiple distinct reasons were labeled “Multiple reasons, for manual review” and adjudicated to select a primary category. Empty termination fields were labeled “Reason not provided, for manual review.” PubMed searches were performed when registry explanations were unavailable.

**Variables Extracted**

Variables included allocation, intervention model, blinding, primary purpose, and endpoint type, intervention type, anticipated enrollment, age eligibility, and sex eligibility. Sample size categories were defined as small (1–100), medium (101–1000), and large (>1000). Age groups were categorized as adults including older adults (≥18 years, without exclusion of ≥65 years), adults only (<65 years, explicitly excluding older adults), older adults only (≥65 years), or other. Sex eligibility was coded as female-only, male-only, or mixed. Funding source was inferred from lead sponsor and collaborator fields. Entities were categorized as industry, university/hospital, NIH/US federal, non-US government, nonprofit/research institute, mixed, or other using an AI-assisted workflow with deterministic settings, caching, and a second-pass verification for ambiguous classifications. Entities not reliably classified were adjudicated manually.

**Recruitment Adequacy**

Recruitment adequacy was defined as the ratio of actual enrollment to anticipated enrollment. Adequate recruitment was ≥ 80% of anticipated enrollment; low recruitment was < 80%.

**Validation and Handling of Potential AI Error**

A random 2% sample (n=506) underwent independent dual-review using a standardized protocol. Reviewers (N.S.C.C. and S.W.C.) verified eligibility, status, endpoint type, termination reason, and enrollment values. Agreement exceeded 98%. Discrepancies were resolved by consensus and corrected in the master dataset. All trials labeled Manual Review by the AI were adjudicated. Following this process, 19,191 trials were finalized for analysis.

**Statistical Analysis**

Trial characteristics were summarized using frequencies and proportions. Termination rates were compared across subgroups using Pearson’s chi-squared test. Multivariable logistic regression estimated adjusted odds ratios with 95% confidence intervals for predictors of termination. Covariates included sponsor type, trial phase, sample size, intervention type, randomization, blinding, primary endpoint, study design, age group, sex, and clinical indication. Low recruitment was modeled both as investigator-reported and objectively defined. Statistical significance was set at p<0.05. All analyses were performed using Stata 14 MP (StataCorp LLC, College Station, TX) and Microsoft Excel (Microsoft Corp, Redmond, WA).

**AI Workflow Transparency**

Exact prompts, category definitions, and executable Python scripts are provided in Supplementary Appendices A and B. The workflow used deterministic settings, structured outputs, automated validation, retry logic, and mandatory manual adjudication for ambiguous outputs to ensure reproducibility and minimize AI-related error.

**eFigure.** PRISMA Flowchart


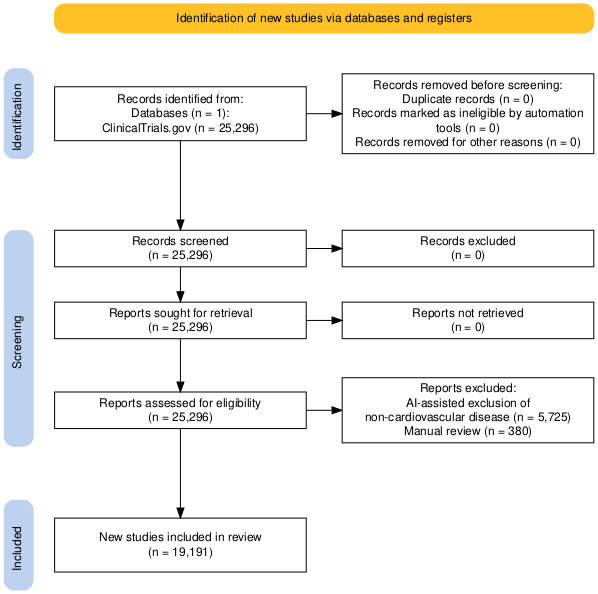


**eTable 1. Status of Completion of Clinical Trials, by Year.**

| Year | Terminated | Completed | Total | Terminated Percentage |
| --- | --- | --- | --- | --- |
| 2000 | 1 | 34 | 35 | 2.86% |
| 2001 | 5 | 47 | 52 | 9.62% |
| 2002 | 2 | 53 | 55 | 3.64% |
| 2003 | 8 | 126 | 134 | 5.97% |
| 2004 | 16 | 194 | 210 | 7.62% |
| 2005 | 27 | 409 | 436 | 6.19% |
| 2006 | 52 | 424 | 476 | 10.92% |
| 2007 | 62 | 535 | 597 | 10.39% |
| 2008 | 79 | 634 | 713 | 11.08% |
| 2009 | 95 | 733 | 828 | 11.47% |
| 2010 | 106 | 674 | 780 | 13.59% |
| 2011 | 103 | 747 | 850 | 12.12% |
| 2012 | 119 | 795 | 914 | 13.02% |
| 2013 | 113 | 827 | 940 | 12.02% |
| 2014 | 144 | 906 | 1050 | 13.71% |
| 2015 | 126 | 955 | 1081 | 11.66% |
| 2016 | 119 | 1011 | 1130 | 10.53% |
| 2017 | 136 | 1019 | 1155 | 11.77% |
| 2018 | 139 | 1040 | 1179 | 11.79% |
| 2019 | 107 | 1138 | 1245 | 8.59% |
| 2020 | 155 | 878 | 1033 | 15.00% |
| 2021 | 135 | 880 | 1015 | 13.30% |
| 2022 | 141 | 999 | 1140 | 12.37% |
| 2023 | 114 | 1032 | 1146 | 9.95% |
| 2024 | 83 | 770 | 853 | 9.73% |
| 2025 | 14 | 80 | 94 | 14.89% |
| **TOTAL** | **2202** | **16989** | **19191** | **11.47%** |
| The table displays the total number of trials registered each year, the number completed, the number terminated, and the corresponding percentage of terminated trials. The overall premature termination rate across all years was 11.47%, with a peak observed in 2020 likely reflecting the impact of the COVID-19 pandemic. | | | | |

**eTable 2. Proportion of Low Recruitment among Prematurely Terminated Trials, by Reported Reason for Termination.**

|  | **Reported low recruitment (Ref)** | **Safety/ Ethics** | **Financial/ Administrative** | **Sponsor/  Business** | **Treatment withdrawn  from market** | **Futility** | **Study Competition/  Replaced** | **Benefit** | **COVID-19  pandemic** | **Other specified  reasons** | **No clear reason for termination** | TOTAL |
| --- | --- | --- | --- | --- | --- | --- | --- | --- | --- | --- | --- | --- |
| **Comorbidity** | | | | | | | | | | | | |
| *Multiple (Ref) | 38.25% | 6.01% | 13.66% | 10.38% | 3.83% | 10.38% | 1.64% | 2.19% | 3.28% | 4.92% | 5.46% | 183 |
| *Hypertension | 43.21% | 3.70% | 11.73% | 12.96% | 0.00% | 4.94% | 2.47% | 1.23% | 3.70% | 5.56% | 10.49% | 162 |
| *Heart Failure/ Cardiomyopathy | 43.11% | 5.28% | 12.90% | 12.32% | 1.47% | 8.21% | 2.64% | 2.05% | 3.23% | 2.93% | 5.87% | 341 |
| *Coronary Artery Disease/  Acute Coronary Syndrome | 47.87% | 4.27% | 9.15% | 11.28% | 1.83% | 7.62% | 0.91% | 0.91% | 2.74% | 1.52% | 11.89% | 328 |
| *Arrhythmias, incl Atrial fibrillation | 50.79% | 5.29% | 5.29% | 12.70% | 0.00% | 8.47% | 1.59% | 1.59% | 1.59% | 2.12% | 10.58% | 189 |
| *Stroke | 30.95% | 6.35% | 13.49% | 6.35% | 0.79% | 12.30% | 3.57% | 5.16% | 2.78% | 8.73% | 9.52% | 252 |
| *Others | 43.91% | 5.49% | 8.84% | 13.79% | 2.54% | 7.90% | 2.81% | 2.14% | 2.81% | 2.95% | 6.83% | 747 |
| **Randomization** | | | | | | | | | | | | |
| *Randomized (Ref) | 44.95% | 5.96% | 10.48% | 9.48% | 1.44% | 9.54% | 2.07% | 2.45% | 2.07% | 3.45% | 8.10% | 1593 |
| *Nonrandomized | 37.90% | 3.51% | 10.02% | 18.36% | 2.67% | 5.51% | 3.17% | 1.50% | 5.01% | 4.34% | 8.01% | 599 |
| *Not specified | 23.08% | 7.69% | 7.69% | 0.00% | 7.69% | 0.00% | 0.00% | 0.00% | 0.00% | 23.08% | 30.77% | 13 |
| **Endpoints** | | | | | | | | | | | | |
| *Safety and Efficacy (Ref) | 41.98% | 5.56% | 8.02% | 15.12% | 3.09% | 9.26% | 1.85% | 1.54% | 5.25% | 3.09% | 5.25% | 324 |
| *Efficacy only | 43.56% | 5.35% | 10.20% | 11.05% | 1.62% | 8.30% | 2.53% | 2.74% | 2.11% | 4.15% | 8.37% | 1421 |
| *Safety only | 35.57% | 7.38% | 8.05% | 20.81% | 1.34% | 11.41% | 2.68% | 0.00% | 4.70% | 1.34% | 6.71% | 149 |
| *Pharmacokinetic/Pharmacodynamic | 45.98% | 4.02% | 14.37% | 8.05% | 1.72% | 5.75% | 2.87% | 1.72% | 2.87% | 3.45% | 9.20% | 174 |
| *Others | 47.90% | 2.52% | 15.97% | 7.56% | 0.84% | 8.40% | 0.84% | 0.84% | 3.36% | 3.36% | 8.40% | 119 |
| *Not stated | 6.67% | 6.67% | 6.67% | 13.33% | 0.00% | 6.67% | 0.00% | 0.00% | 0.00% | 0.00% | 60.00% | 15 |
| **Study Design** | | | | | | | | | | | | |
| *Parallel (Ref) | 44.92% | 6.03% | 9.96% | 9.82% | 1.42% | 9.49% | 1.76% | 2.64% | 1.96% | 4.13% | 7.86% | 1476 |
| *Cross-over | 47.62% | 1.90% | 13.33% | 10.48% | 2.86% | 4.76% | 4.76% | 0.00% | 4.76% | 1.90% | 7.62% | 105 |
| *Factorial | 44.74% | 10.53% | 13.16% | 2.63% | 2.63% | 10.53% | 2.63% | 2.63% | 2.63% | 5.26% | 2.63% | 38 |
| *Single arm | 37.99% | 3.76% | 10.57% | 18.10% | 2.51% | 5.73% | 3.58% | 1.43% | 5.02% | 2.33% | 8.96% | 558 |
| *Sequential | 15.38% | 0.00% | 7.69% | 23.08% | 0.00% | 23.08% | 0.00% | 0.00% | 0.00% | 23.08% | 7.69% | 13 |
| *Not available | 16.67% | 0.00% | 16.67% | 8.33% | 0.00% | 16.67% | 0.00% | 0.00% | 0.00% | 0.00% | 41.67% | 12 |
| **Blinding** | | | | | | | | | | | | |
| *Open label (Ref) | 46.02% | 4.11% | 10.81% | 13.76% | 2.41% | 2.41% | 2.14% | 3.31% | 3.84% | 3.84% | 7.33% | 1119 |
| *Single blind | 48.40% | 3.85% | 11.86% | 9.29% | 2.24% | 2.24% | 3.21% | 2.88% | 5.13% | 5.13% | 5.77% | 312 |
| *Double blind | 40.78% | 8.63% | 9.41% | 11.37% | 0.78% | 1.18% | 1.18% | 4.31% | 2.75% | 2.75% | 16.86% | 255 |
| *Triple/Quadruple blind | 43.10% | 8.87% | 11.33% | 12.07% | 0.74% | 3.69% | 2.71% | 1.48% | 3.69% | 3.69% | 8.62% | 406 |
| *Not available | 20.00% | 0.00% | 0.00% | 20.00% | 0.00% | 0.00% | 0.00% | 0.00% | 0.00% | 0.00% | 60.00% | 5 |
| **Purpose** | | | | | | | | | | | | |
| *Treatment (Ref) | 41.67% | 6.14% | 9.30% | 12.73% | 1.42% | 8.72% | 2.65% | 2.39% | 2.78% | 3.23% | 8.98% | 1548 |
| *Diagnosis | 55.06% | 1.27% | 9.49% | 9.49% | 2.53% | 4.43% | 1.27% | 0.00% | 5.70% | 1.90% | 8.86% | 158 |
| *Prevention | 41.70% | 5.38% | 14.80% | 7.62% | 2.24% | 13.90% | 0.45% | 2.24% | 2.24% | 2.69% | 6.73% | 223 |
| *Other/ Unclassified | 44.32% | 2.56% | 13.19% | 12.09% | 2.93% | 4.76% | 2.93% | 2.20% | 2.20% | 8.06% | 4.76% | 273 |
| **Intervention** | | | | | | | | | | | | |
| *Drug (Ref) | 42.30% | 7.45% | 8.66% | 11.98% | 1.11% | 11.38% | 2.01% | 2.32% | 1.71% | 1.91% | 9.16% | 993 |
| *Device | 40.50% | 4.20% | 10.59% | 15.46% | 3.53% | 6.22% | 3.03% | 2.52% | 4.03% | 3.36% | 6.55% | 595 |
| *Biologics | 28.57% | 5.71% | 20.00% | 17.14% | 0.00% | 8.57% | 2.86% | 0.00% | 2.86% | 0.00% | 14.29% | 35 |
| *Behavior/Diet/Lifestyle | 44.52% | 2.05% | 16.44% | 7.53% | 0.68% | 3.42% | 2.05% | 0.00% | 2.74% | 10.96% | 9.59% | 146 |
| *Others/Unspecified | 48.50% | 2.77% | 11.09% | 7.85% | 1.39% | 6.47% | 2.31% | 2.31% | 3.93% | 6.00% | 7.39% | 433 |
| **Trial Size** | | | | | | | | | | | | |
| *1-100 | 32.95% | 9.38% | 8.70% | 9.61% | 2.29% | 18.76% | 2.29% | 3.66% | 2.29% | 2.06% | 8.01% | 437 |
| *101-1000 (Ref) | 47.51% | 3.54% | 11.02% | 11.98% | 1.62% | 5.03% | 2.40% | 1.38% | 3.00% | 4.31% | 8.21% | 1669 |
| *>1000 | 5.81% | 17.44% | 5.81% | 20.93% | 2.33% | 23.26% | 2.33% | 10.47% | 3.49% | 0.00% | 8.14% | 86 |
| *Not available | 40.00% | 10.00% | 10.00% | 20.00% | 0.00% | 0.00% | 0.00% | 0.00% | 0.00% | 0.00% | 20.00% | 10 |
| **Participation by Gender** | | | | | | | | | | | | |
| *Both males and females (Ref) | 42.54% | 5.33% | 10.33% | 12.16% | 1.82% | 8.46% | 2.43% | 2.24% | 2.81% | 3.74% | 8.13% | 2139 |
| *All females | 43.48% | 4.35% | 8.70% | 4.35% | 0.00% | 17.39% | 0.00% | 0.00% | 0.00% | 0.00% | 21.74% | 23 |
| *All males | 65.00% | 2.50% | 12.50% | 2.50% | 0.00% | 2.50% | 0.00% | 0.00% | 7.50% | 2.50% | 5.00% | 40 |
| **Participation by Age Group** | | | | | | | | | | | | |
| *Adults, including Senior Citizens (Ref) | 42.96% | 4.96% | 10.67% | 12.22% | 1.75% | 8.36% | 2.30% | 2.30% | 2.85% | 3.81% | 7.81% | 1997 |
| *Adults only | 48.28% | 1.72% | 13.79% | 8.62% | 1.72% | 1.72% | 5.17% | 0.00% | 1.72% | 3.45% | 13.79% | 58 |
| *Senior Citizens only | 50.00% | 11.11% | 8.33% | 11.11% | 2.78% | 11.11% | 0.00% | 0.00% | 0.00% | 0.00% | 5.56% | 36 |
| *Others, including children | 37.84% | 10.81% | 3.60% | 8.11% | 1.80% | 12.61% | 2.70% | 1.80% | 4.50% | 2.70% | 13.51% | 111 |
| **Sponsor** | | | | | | | | | | | | |
| *Industry (Ref) | 29.37% | 6.73% | 5.16% | 23.78% | 1.43% | 11.75% | 3.44% | 2.44% | 3.30% | 1.15% | 11.46% | 698 |
| *NIH | 31.43% | 5.71% | 17.14% | 2.86% | 2.86% | 11.43% | 5.71% | 2.86% | 5.71% | 2.86% | 11.43% | 35 |
| *University Hospital | 52.94% | 3.71% | 13.04% | 4.86% | 2.56% | 5.50% | 2.17% | 2.69% | 1.92% | 4.22% | 6.39% | 782 |
| *Mixed source funding | 44.74% | 5.45% | 12.41% | 8.65% | 0.94% | 8.27% | 1.32% | 1.50% | 3.20% | 6.58% | 6.95% | 532 |
| *Others | 50.82% | 6.56% | 9.84% | 8.20% | 1.64% | 4.92% | 1.64% | 0.00% | 4.92% | 3.28% | 8.20% | 61 |
| *Non-US Government | 54.55% | 0.00% | 13.64% | 0.00% | 4.55% | 18.18% | 0.00% | 0.00% | 4.55% | 0.00% | 4.55% | 22 |
| *Research Institutes/  Nonprofit organization | 48.61% | 6.94% | 12.50% | 8.33% | 1.39% | 8.33% | 1.39% | 1.39% | 2.78% | 2.78% | 5.56% | 72 |
| **Phase of Trial** | | | | | | | | | | | | |
| *Not applicable (Ref) | 31.85% | 2.47% | 8.87% | 7.71% | 1.75% | 3.93% | 1.96% | 1.31% | 4.15% | 31.85% | 4.15% | 1375 |
| *Phases 0 (early phase 1)/1 | 28.57% | 5.00% | 9.29% | 7.86% | 0.71% | 4.29% | 1.43% | 1.43% | 1.43% | 28.57% | 11.43% | 140 |
| *Phases 1/2 or 2 | 26.06% | 4.65% | 8.89% | 13.13% | 0.40% | 6.67% | 1.01% | 1.41% | 2.63% | 26.06% | 9.09% | 495 |
| *Phases 2/3 or 3 | 26.53% | 7.06% | 3.82% | 9.92% | 0.76% | 13.36% | 1.91% | 2.86% | 0.95% | 26.53% | 6.30% | 524 |
| *Phase 4 | 36.30% | 2.72% | 5.26% | 5.08% | 1.45% | 4.17% | 1.45% | 1.09% | 0.73% | 36.30% | 5.44% | 551 |
| Trials were categorized based on whether low recruitment was cited exclusively or in combination with other reasons. Percentages in the table indicate the proportion of low recruitment cited for each reason. This analysis highlights patterns and predictors of enrollment-related trial failure across subgroups. | | | | | | | | | | | | |

**Supplementary Appendix A. AI-Assisted Screening and Classification Methodology**

This appendix describes the methodological framework for AI-assisted screening, classification, and categorization of cardiovascular clinical trials retrieved from ClinicalTrials.gov. The corresponding executable Python scripts are provided in **Supplementary Appendix B** to support full reproducibility.

All scripts were executed locally using Python ≥3.8 and the OpenAI API with deterministic settings (temperature = 0.0). No external databases, internet searches, or external knowledge retrieval were used by the AI models.

**Overview of the AI-Assisted Workflow**

The AI-assisted pipeline consisted of three sequential components:

**A1. Cardiovascular trial relevance screening**
**A2. Trial termination reason categorization**
**A3. Sponsor funding source categorization**

Each component was implemented using structured prompt design, JSON-only output enforcement, automated validation, retry logic, and periodic progress saving.

All AI outputs were subsequently subjected to independent manual validation.

**Software Environment**

All analyses were performed using Python ≥3.8 with the following packages:

- pandas
- openai
- tqdm
- tkinter
- numpy

**A1. Cardiovascular Trial Relevance Screening**

**Objective:** To classify each trial as **Include**, **Exclude**, or **Manual Review** based on cardiovascular relevance using trial title and condition fields.

**Approach:** Trials were processed in small batches. For each batch, the AI model received structured JSON input and returned classification decisions in strict JSON format. Cardiovascular relevance was defined using comprehensive inclusion and exclusion criteria covering cardiac, vascular, and cerebrovascular diseases.

**Quality Controls:**

- JSON-only output enforcement
- Automatic response structure validation
- Batch-level completeness checks
- Retry logic with exponential backoff
- Automatic labeling of failed batches for re-processing

**A2. Trial Termination Reason Categorization**

**Objective:** To convert free-text termination reasons into standardized categories.

**Predefined Categories:**

- Low recruitment
- Safety or ethics issue
- Sponsor or business decisions
- Financial or administrative reasons
- Futility
- Benefit
- Study competition or replacement
- Treatment withdrawn from market
- Multiple reasons, for manual review
- Other, for manual review

**Approach:** Each termination reason was processed individually using deterministic AI classification with strict category enforcement. Only one category was allowed per record.

**Special Handling:**

- Empty or missing reasons were automatically labeled for manual review.
- API-derived placeholder texts were assigned predefined manual-review categories.
- Unexpected AI outputs were automatically flagged.

**A3. Sponsor Funding Source Categorization**

**Objective:** To classify sponsors and collaborators into standardized funding source categories.

**Funding Categories:**

- Industry
- NIH / US Federal
- University / Hospital
- Government
- Research institute / Non-profit organization
- Other
- Mixed source

**Hybrid Classification Strategy:**

1. Deterministic AI classification of each entity
2. Local caching to avoid duplicate AI calls
3. Rule-based consolidation across sponsors and collaborators
4. Automatic identification of mixed funding sources

A two-pass verification system was applied when the initial classification was ambiguous.

**Reproducibility and Validation Procedures**

To ensure reproducibility and methodological rigor:

- All AI calls used temperature = 0.0.
- JSON-only output formatting was enforced.
- API failures triggered automated retry logic with exponential backoff.
- Intermediate outputs were saved periodically to prevent data loss.
- All AI outputs were independently reviewed by two investigators.
- Discrepancies were resolved by consensus review.

**Data Outputs**

Each component generated structured Excel outputs containing:

- AI screening decisions
- AI-categorized termination reasons
- Sponsor funding source classifications

These derived datasets were used for all downstream statistical analyses.

**Availability of Full Scripts**

The complete executable Python scripts for all three components are provided in **Supplementary Appendix B** to support full transparency and reproducibility.

**Supplementary Appendix B. Full Python Scripts**

This appendix provides the complete executable Python scripts used for AI-assisted screening, classification, and categorization of cardiovascular clinical trials as described in Supplementary Appendix A.

**B1. Cardiovascular Trial Relevance Screening**

```python

import tkinter as tk

from tkinter import filedialog, messagebox

import pandas as pd

import openai

import os

import time

import logging

import json

# --- CONFIGURATION CONSTANTS ---

API_KEY_ENV_VAR = "OPENAI_API_KEY"

BATCH_SIZE = 10

# OpenAI Model

REQUESTED_MODEL = "gpt-4.1-mini"

FALLBACK_MODEL_1 = "gpt-4o-mini"

FALLBACK_MODEL_2 = "gpt-3.5-turbo" # Ensure this model supports JSON mode if used

# Column Names

TITLE_COLUMN = "Study Title"

CONDITIONS_COLUMN = "Conditions"

DECISION_COLUMN = "AI_Decision"

# Output File Suffix

OUTPUT_FILE_SUFFIX = "_classified_batched"

# API Call Parameters

MAX_RETRIES = 3

RETRY_DELAY_SECONDS = 5

INTER_REQUEST_DELAY_SECONDS = 1

# Progress Saving

SAVE_EVERY_N_ROWS = 50

# Logging Configuration

LOG_FILE_NAME = "cardiovascular_trial_classification.log"

LOG_LEVEL = logging.INFO

# Classification Labels

LABEL_INCLUDE = "Include"

LABEL_EXCLUDE = "Exclude"

LABEL_MANUAL_REVIEW = "Manual Review"

LABEL_ERROR_RETRY_LATER = "Screening Error - Retry Later"

# --- GLOBAL VARIABLES ---

current_openai_model = REQUESTED_MODEL

# --- LOGGING SETUP ---

def setup_logging():

"""Configures logging for the script."""

logging.basicConfig(

level=LOG_LEVEL,

format="%(asctime)s - %(levelname)s - %(message)s",

handlers=[logging.FileHandler(LOG_FILE_NAME), logging.StreamHandler()]

)

logging.info("Logging initialized.")

# --- PROMPT ENGINEERING FOR BATCHES ---

def get_batch_classification_prompt(batch_df: pd.DataFrame) -> str:

"""Generates the detailed prompt for a batch of trials."""

trials_json_list = []

for index, row in batch_df.iterrows():

trials_json_list.append({

"id": index,

"title": str(row[TITLE_COLUMN]),

"conditions": str(row[CONDITIONS_COLUMN])

})

trials_json_string = json.dumps(trials_json_list, indent=2)

return f"""You are an expert assistant classifying clinical trials for cardiovascular relevance.

**Instructions:**

1. Process each clinical trial object in the input JSON array below.

2. For each trial, analyze its 'title' and 'conditions'.

3. Decide if the trial should be 'Include', 'Exclude', or marked for 'Manual Review' based on the criteria.

4. Return a single JSON object. This object must contain one key, "decisions", whose value is an array of objects.

5. Each object in the "decisions" array must have two keys: the original 'id' (as an integer) and your 'decision' (as a string).

**Inclusion Criteria (Cardiovascular Diseases):**

Pathologic conditions involving the heart, blood vessels, or pericardium. This includes cardiovascular abnormalities, infections, heart diseases (cardiomyopathies, valve diseases, ischemia, arrhythmias, etc.), and vascular diseases (aneurysms, thrombosis, hypertension, cerebrovascular disorders, etc.). All trials involving stroke should be included.

**Exclusion Criteria:**

- Trials not related to the cardiovascular system as defined above.

**Decision Categories:**

- '{LABEL_INCLUDE}': The trial clearly fits the criteria.

- '{LABEL_EXCLUDE}': The trial clearly does NOT fit the criteria.

- '{LABEL_MANUAL_REVIEW}': Use this sparingly, only for significant ambiguity.

**Input Trials JSON:**

{trials_json_string}

**Provide your output in the specified JSON format only.**

"""

# --- OPENAI API BATCH INTERACTION ---

def classify_batch_with_llm(client: openai.OpenAI, batch_df: pd.DataFrame) -> dict:

"""Calls the OpenAI API to classify a batch of trials and returns a dictionary of decisions."""

prompt = get_batch_classification_prompt(batch_df)

attempt = 0

while attempt < MAX_RETRIES:

try:

completion = client.chat.completions.create(

model=current_openai_model,

messages=[

{"role": "system", "content": "You are a helpful assistant that processes clinical trial data and returns responses in JSON format."},

{"role": "user", "content": prompt}

],

response_format={"type": "json_object"},

temperature=0.0

)

response_text = completion.choices[0].message.content

response_json = json.loads(response_text)

# --- Validation of Response ---

if 'decisions' not in response_json or not isinstance(response_json['decisions'], list):

logging.warning("API response is missing 'decisions' array. Retrying...")

raise ValueError("Invalid JSON structure from API")

decisions_map = {}

for item in response_json['decisions']:

if 'id' in item and 'decision' in item:

decisions_map[item['id']] = item['decision']

else:

logging.warning(f"Skipping malformed decision object: {item}")

# Check if all original IDs were returned

if len(decisions_map) != len(batch_df):

logging.warning(f"API returned {len(decisions_map)} decisions for a batch of {len(batch_df)}. Retrying...")

raise ValueError("Mismatch in decision count from API")

return decisions_map

except Exception as e:

logging.error(f"Error on attempt {attempt + 1}/{MAX_RETRIES} for batch starting at index {batch_df.index[0]}: {e}")

attempt += 1

time.sleep(RETRY_DELAY_SECONDS * (2 ** attempt)) # Exponential backoff

if attempt == MAX_RETRIES:

return {index: LABEL_ERROR_RETRY_LATER for index in batch_df.index}

return {}

# --- FILE HANDLING AND MAIN PROCESSING LOGIC ---

def process_trials(df: pd.DataFrame, client: openai.OpenAI, output_file_path: str):

"""Iterates through the DataFrame in batches, classifies trials, and saves progress."""

# Determine rows to process

unprocessed_indices = df[~df[DECISION_COLUMN].isin([LABEL_INCLUDE, LABEL_EXCLUDE, LABEL_MANUAL_REVIEW])].index

logging.info(f"Found {len(unprocessed_indices)} total rows to process.")

processed_in_session = 0

# Process in batches

for i in range(0, len(unprocessed_indices), BATCH_SIZE):

batch_indices = unprocessed_indices[i:i + BATCH_SIZE]

if len(batch_indices) == 0:

continue

batch_df = df.loc[batch_indices]

logging.info(f"Processing batch of {len(batch_df)} trials (starting at row {batch_indices[0] + 2})...")

decisions = classify_batch_with_llm(client, batch_df)

# Update the main DataFrame with decisions from the batch

for index, decision in decisions.items():

df.loc[index, DECISION_COLUMN] = decision

processed_in_session += len(batch_df)

if processed_in_session % SAVE_EVERY_N_ROWS < BATCH_SIZE and processed_in_session > SAVE_EVERY_N_ROWS:

try:

df.to_excel(output_file_path, index=False)

logging.info(f"Progress saved to {output_file_path}.")

except Exception as e:

logging.error(f"Error saving progress to Excel: {e}")

time.sleep(INTER_REQUEST_DELAY_SECONDS)

# Final save

try:

df.to_excel(output_file_path, index=False)

logging.info(f"All batches processed. Final data saved to {output_file_path}.")

except Exception as e:

logging.error(f"Error on final save: {e}")

# --- MAIN EXECUTION ---

def main():

"""Main function to orchestrate the classification process."""

setup_logging()

# (The rest of the main function remains largely the same: GUI selection, loading files, etc.)

# ...

# Initialize OpenAI Client

api_key = os.getenv(API_KEY_ENV_VAR)

if not api_key:

messagebox.showerror("API Key Error", f"Environment variable {API_KEY_ENV_VAR} not set.")

return

client = openai.OpenAI(api_key=api_key)

# Select and Load File

input_file_path = filedialog.askopenfilename(title="Select Input Excel File")

if not input_file_path:

logging.info("No file selected. Exiting.")

return

try:

df = pd.read_excel(input_file_path)

except Exception as e:

messagebox.showerror("File Read Error", f"Could not read Excel file: {e}")

return

# Prepare DataFrame

base, ext = os.path.splitext(input_file_path)

output_file_path = f"{base}{OUTPUT_FILE_SUFFIX}{ext}"

if DECISION_COLUMN not in df.columns:

df[DECISION_COLUMN] = ""

df[DECISION_COLUMN] = df[DECISION_COLUMN].astype(str).replace('nan', '')

# --- Initial Processing Pass ---

logging.info("--- Starting Batch Processing Pass ---")

process_trials(df, client, output_file_path)

# --- Retry Pass for Errors ---

if df[DECISION_COLUMN].isin([LABEL_ERROR_RETRY_LATER]).any():

logging.info("--- Starting Retry Pass for Failed Batches ---")

process_trials(df, client, output_file_path) # The same function will pick up unprocessed rows

else:

logging.info("No batches marked for retry.")

# Final check

remaining_errors = df[df[DECISION_COLUMN] == LABEL_ERROR_RETRY_LATER].shape[0]

if remaining_errors > 0:

messagebox.showwarning("Processing Incomplete", f"{remaining_errors} trial(s) failed all processing attempts.")

else:

messagebox.showinfo("Processing Complete", f"All trials processed. Results saved to {output_file_path}")

if __name__ == "__main__":

main()

**B2. Trial Termination Reason Categorization**

```python

import tkinter as tk

from tkinter import filedialog

import pandas as pd

import openai

import os

import time

from tqdm import tqdm # For progress bar

import numpy as np # For pd.NA if preferred

# --- Configuration ---

API_RETRY_LIMIT = 3

API_RETRY_DELAY = 5 # Initial delay in seconds for retries

# BATCH_SIZE is implicitly 1 for LLM calls, tqdm will show item progress.

# Saving progress will be based on number of items processed by LLM.

SAVE_EVERY_N_ITEMS_FOR_LLM = 50 # Save progress after every N items processed by LLM. Adjust as needed.

OUTPUT_FILENAME = "categorized_trials_output_v2.xlsx" # Changed filename slightly for distinction

INPUT_COLUMN_NAME = "reason_for_termination"

OUTPUT_COLUMN_NAME = "reason_for_termination_categorized"

MODEL_NAME = "gpt-4.1-mini" # As specified by user

# Specific text to check in input and the output to assign

SPECIFIC_INPUT_REASON_TO_EXCLUDE = "Manual Review Required (Terminated, but 'whyStopped' field empty/missing in API data)"

SPECIFIC_OUTPUT_FOR_EXCLUDED_REASON = "Manual review required, reason unavailable in original API"

# --- OpenAI API Key ---

try:

client = openai.OpenAI(api_key=os.environ.get("OPENAI_API_KEY"))

if not client.api_key:

raise ValueError("OPENAI_API_KEY environment variable not set or empty.")

except Exception as e:

print(f"Error initializing OpenAI client: {e}")

print("Please ensure the OPENAI_API_KEY environment variable is correctly set.")

exit()

# --- LLM Prompt and Categories ---

CATEGORIES_FOR_LLM = [

"Low recruitment",

"Safety or ethics issue",

"Sponsor or business decisions",

"Financial or admin reasons",

"Futility (trial stopped due to futility)",

"Benefit (trial stopped because results show a clear advantage for one treatment arm over the control group)",

"Study competition or replaced for another study",

"Treatment withdrawn from market",

"Multiple reasons, for manual review",

"Other, for manual review"

]

SYSTEM_PROMPT = f"""

You are an expert assistant tasked with categorizing clinical trial termination reasons.

Given a reason for trial termination, you must categorize it into *exactly one* of the following predefined categories:

{', '.join([f'"{cat}"' for cat in CATEGORIES_FOR_LLM])}

Guidelines for categorization:

- If the provided reason clearly indicates multiple distinct factors that would otherwise fall into different primary categories (e.g., both safety and low recruitment), you MUST categorize it as "Multiple reasons, for manual review".

- If the reason is singular but does not clearly fit into any of the other specific categories, you MUST categorize it as "Other, for manual review".

- Otherwise, select the most appropriate specific category.

Respond ONLY with the category name from the list above. Do not add any explanations, numbering, or extraneous text.

For example, if the reason is "Study closed due to poor enrollment", you should respond:

Low recruitment

"""

# --- Helper Functions ---

def select_file():

"""Opens a dialog for file selection and returns the path."""

root = tk.Tk()

root.withdraw() # Hide the main tkinter window

file_path = filedialog.askopenfilename(

title="Select Excel File",

filetypes=(("Excel files", "*.xlsx"), ("All files", "*.*"))

)

return file_path

def get_llm_category(reason_text_for_llm):

"""

Gets category for a single reason text using OpenAI API with retry logic.

This function should only be called for reasons that genuinely need LLM processing.

"""

# Basic check, though main() should filter most problematic cases before calling this

if not reason_text_for_llm or pd.isna(reason_text_for_llm) or reason_text_for_llm.strip() == "":

# This case should ideally be caught by the preprocessing loop in main()

return "Reason not provided, for manual review"

retries = 0

while retries < API_RETRY_LIMIT:

try:

response = client.chat.completions.create(

model=MODEL_NAME,

messages=[

{"role": "system", "content": SYSTEM_PROMPT},

{"role": "user", "content": reason_text_for_llm}

],

temperature=0.0, # For deterministic categorization

max_tokens=50 # Max tokens for category name

)

category = response.choices[0].message.content.strip()

if category in CATEGORIES_FOR_LLM:

return category

else:

# If LLM returns something unexpected

tqdm.write(f"Warning: LLM returned an unexpected category '{category}' for reason: '{reason_text_for_llm}'. Tagging as 'Other, for manual review'.")

return "Other, for manual review"

except openai.APIError as e:

tqdm.write(f"OpenAI API Error for reason '{reason_text_for_llm}': {e}. Retrying in {API_RETRY_DELAY * (2**retries)}s...")

time.sleep(API_RETRY_DELAY * (2**retries))

retries += 1

except Exception as e: # Catch other potential errors like network issues

tqdm.write(f"An unexpected error occurred with the API call for reason '{reason_text_for_llm}': {e}. Retrying in {API_RETRY_DELAY * (2**retries)}s...")

time.sleep(API_RETRY_DELAY * (2**retries))

retries += 1

tqdm.write(f"Failed to categorize reason after {API_RETRY_LIMIT} retries: '{reason_text_for_llm}'")

return "Failed to categorize, needs re-processing"

# --- Main Processing Logic ---

def main():

tqdm.write("Starting trial termination categorization process...")

file_path = select_file()

if not file_path:

tqdm.write("No file selected. Exiting.")

return

tqdm.write(f"Selected file: {file_path}")

try:

df = pd.read_excel(file_path)

tqdm.write(f"Successfully loaded data. Found {len(df)} rows.")

except Exception as e:

tqdm.write(f"Error loading Excel file: {e}")

return

if INPUT_COLUMN_NAME not in df.columns:

tqdm.write(f"Error: Input column '{INPUT_COLUMN_NAME}' not found in the Excel file.")

tqdm.write(f"Available columns are: {df.columns.tolist()}")

return

# --- Resume Logic & Output Column Initialization ---

if OUTPUT_COLUMN_NAME not in df.columns:

tqdm.write(f"Output column '{OUTPUT_COLUMN_NAME}' not found, creating it.")

df[OUTPUT_COLUMN_NAME] = pd.NA # Use pd.NA for missing values if desired, or ""

else:

tqdm.write(f"Output column '{OUTPUT_COLUMN_NAME}' found. Will attempt to resume.")

# Ensure output column is string type for consistent checking, fill NA/NaN with empty string for checks

df[OUTPUT_COLUMN_NAME] = df[OUTPUT_COLUMN_NAME].astype(str).replace('nan', '').replace('<NA>', '').fillna('')

reasons_for_llm_processing = []

indices_for_llm_processing = []

rows_updated_in_preprocessing = 0

# --- Preprocessing Pass ---

# Identify rows that need processing or can be directly assigned.

tqdm.write("Preprocessing reasons (checking for existing data and special cases)...")

for index, row in tqdm(df.iterrows(), total=df.shape[0], desc="Preprocessing Rows"):

input_reason = row[INPUT_COLUMN_NAME]

# Ensure input_reason is a string for consistent comparisons, handle NaN/None

if pd.isna(input_reason):

input_reason_str = ""

else:

input_reason_str = str(input_reason).strip()

current_category = str(row[OUTPUT_COLUMN_NAME]).strip()

# 1. Skip if already validly categorized

if current_category != "" and current_category != "Failed to categorize, needs re-processing":

continue # Already processed and valid, skip.

# 2. Handle the SPECIFIC user-defined exclusion rule (this will NOT go to LLM)

if input_reason_str == SPECIFIC_INPUT_REASON_TO_EXCLUDE:

if df.loc[index, OUTPUT_COLUMN_NAME] != SPECIFIC_OUTPUT_FOR_EXCLUDED_REASON:

df.loc[index, OUTPUT_COLUMN_NAME] = SPECIFIC_OUTPUT_FOR_EXCLUDED_REASON

rows_updated_in_preprocessing +=1

continue # Processed, skip LLM.

# 3. Handle empty or whitespace-only input reasons (this will NOT go to LLM)

if not input_reason_str: # Covers None, NaN (after str conversion to ""), and empty strings

if df.loc[index, OUTPUT_COLUMN_NAME] != "Reason not provided, for manual review":

df.loc[index, OUTPUT_COLUMN_NAME] = "Reason not provided, for manual review"

rows_updated_in_preprocessing +=1

continue # Processed, skip LLM.

# 4. If not skipped or directly assigned, it needs LLM processing

reasons_for_llm_processing.append(input_reason_str)

indices_for_llm_processing.append(index)

if rows_updated_in_preprocessing > 0:

tqdm.write(f"{rows_updated_in_preprocessing} rows were updated during preprocessing (special cases/empty reasons).")

# --- LLM Categorization Pass ---

if not reasons_for_llm_processing:

tqdm.write("No new reasons found requiring LLM categorization.")

else:

tqdm.write(f"\nStarting LLM categorization for {len(reasons_for_llm_processing)} reasons...")

items_processed_count_for_save = 0

for i in tqdm(range(len(reasons_for_llm_processing)), desc="LLM Categorization Progress"):

current_df_index = indices_for_llm_processing[i]

reason_text = reasons_for_llm_processing[i]

# Get category from LLM

category = get_llm_category(reason_text)

# Update DataFrame immediately

df.loc[current_df_index, OUTPUT_COLUMN_NAME] = category

items_processed_count_for_save += 1

# Save progress periodically

if SAVE_EVERY_N_ITEMS_FOR_LLM > 0 and items_processed_count_for_save % SAVE_EVERY_N_ITEMS_FOR_LLM == 0:

try:

df.to_excel(OUTPUT_FILENAME, index=False)

tqdm.write(f"\nProgress saved to '{OUTPUT_FILENAME}'. Processed {i + 1} of {len(reasons_for_llm_processing)} LLM items so far.")

except Exception as e:

tqdm.write(f"\nError saving progress: {e}")

tqdm.write("\nLLM categorization finished for all pending items.")

# --- Final Save ---

tqdm.write("Performing final save of all data...")

try:

df.to_excel(OUTPUT_FILENAME, index=False)

tqdm.write(f"All data successfully saved to '{OUTPUT_FILENAME}'")

except Exception as e:

tqdm.write(f"Error during final save: {e}")

tqdm.write("Process complete.")

if __name__ == "__main__":

main()

**B3. Sponsor Funding Source Categorization**

```python

import tkinter as tk

from tkinter import filedialog, messagebox

import pandas as pd

import os

from openai import OpenAI

import re

from tqdm import tqdm

import time

class SponsorCategorizerApp:

def __init__(self, root):

self.root = root

self.root.title("Cardiovascular Trial Sponsor Categorizer")

self.root.geometry("600x450") # Increased height for better layout

self.client = None

self.setup_openai_client()

self.file_path = None

self.output_file_path = None

# --- REFINEMENT: Caching dictionary to store classifications ---

# This avoids re-classifying the same sponsor/collaborator name multiple times.

self.classification_cache = {}

self.progress_save_interval = 100 # Save progress every 100 rows

self.max_retries = 3

self.create_widgets()

def setup_openai_client(self):

"""Sets up the OpenAI client using the API key from environment variables."""

api_key = os.getenv("OPENAI_API_KEY")

if not api_key:

messagebox.showerror("API Key Error", "OPENAI_API_KEY environment variable not set.")

self.root.destroy()

return

try:

self.client = OpenAI(api_key=api_key)

except Exception as e:

messagebox.showerror("OpenAI Client Error", f"Failed to initialize OpenAI client: {e}")

self.root.destroy()

def create_widgets(self):

"""Creates the GUI widgets."""

main_frame = tk.Frame(self.root, padx=20, pady=20)

main_frame.pack(expand=True, fill=tk.BOTH)

tk.Label(main_frame, text="Select an Excel file for sponsor categorization.", font=("Arial", 12)).pack(pady=10)

self.select_button = tk.Button(main_frame, text="Select Excel File", command=self.select_file, font=("Arial", 10, "bold"), bg="#4CAF50", fg="white")

self.select_button.pack(pady=10)

self.file_label = tk.Label(main_frame, text="No file selected.", font=("Arial", 9), wraplength=550)

self.file_label.pack(pady=5)

self.process_button = tk.Button(main_frame, text="Process and Categorize", command=self.process_file, state=tk.DISABLED, font=("Arial", 10, "bold"))

self.process_button.pack(pady=20)

self.status_label = tk.Label(main_frame, text="Status: Ready", font=("Arial", 9), wraplength=550)

self.status_label.pack(pady=10)

self.output_label = tk.Label(main_frame, text="", font=("Arial", 9, "italic"), fg="blue", wraplength=550)

self.output_label.pack(pady=5)

def select_file(self):

"""Opens a file dialog for selecting an Excel file."""

filetypes = [("Excel files", "*.xlsx *.xls")]

self.file_path = filedialog.askopenfilename(filetypes=filetypes)

if self.file_path:

self.file_label.config(text=f"Selected File: {os.path.basename(self.file_path)}")

self.process_button.config(state=tk.NORMAL)

self.status_label.config(text="Status: File selected. Ready to process.")

self.output_label.config(text="")

else:

self.file_label.config(text="No file selected.")

self.process_button.config(state=tk.DISABLED)

def process_file(self):

"""

Reads the Excel file, processes each row using a hybrid Python + LLM approach,

and saves the results.

"""

if not self.file_path:

messagebox.showwarning("No File", "Please select an Excel file first.")

return

self.status_label.config(text="Status: Preparing to process...")

self.root.update_idletasks()

try:

df = pd.read_excel(self.file_path)

required_cols = ['Lead Sponsor', 'Lead Sponsor Class', 'Collaborators']

if not all(col in df.columns for col in required_cols):

messagebox.showerror("Column Error", f"Missing one or more required columns: {', '.join(required_cols)}")

self.status_label.config(text="Status: Error")

return

if 'Funding Category' not in df.columns:

df['Funding Category'] = ''

dir_name, file_name = os.path.split(self.file_path)

base_name, ext = os.path.splitext(file_name)

temp_file_path = os.path.join(dir_name, f"{base_name}_categorized_temp{ext}")

start_index = 0

if os.path.exists(temp_file_path):

if messagebox.askyesno("Resume Session?", f"A temporary file was found. This may be from a previous run. Do you want to resume from where you left off?"):

resumed_df = pd.read_excel(temp_file_path)

if 'Funding Category' in resumed_df.columns:

df = resumed_df.copy()

# Find the first row that hasn't been processed yet

unprocessed = df[df['Funding Category'].replace('', pd.NA).isna()]

if not unprocessed.empty:

start_index = unprocessed.index[0]

messagebox.showinfo("Resuming Progress", f"Resuming from row {start_index + 1}.")

else:

start_index = len(df) # Already complete

total_rows = len(df)

if start_index >= total_rows:

messagebox.showinfo("Already Completed", "The file appears to be fully categorized already.")

self.status_label.config(text="Status: Complete")

self.output_label.config(text=f"Results are in: {temp_file_path}")

return

with tqdm(total=total_rows, initial=start_index, desc="Processing Rows") as pbar:

for index in range(start_index, total_rows):

row = df.iloc[index]

lead_sponsor = str(row['Lead Sponsor']) if pd.notna(row['Lead Sponsor']) else ''

collaborators_str = str(row['Collaborators']) if pd.notna(row['Collaborators']) else ''

if not lead_sponsor:

df.at[index, 'Funding Category'] = 'Manual Review'

pbar.update(1)

continue

try:

# --- REFINEMENT: Hybrid Logic ---

# 1. Get categories for all involved entities (lead sponsor and collaborators).

entity_categories = set()

lead_sponsor_category = self.get_entity_category(lead_sponsor)

# Edge Case 2: Identify if sponsor is an individual (classified as 'Other').

# If so, their category doesn't count unless they are the only funder.

is_individual_sponsor = lead_sponsor_category == 'Other' and len(lead_sponsor.split()) < 4

collaborator_entities = self.parse_collaborators(collaborators_str)

collaborator_categories = {self.get_entity_category(name) for name in collaborator_entities}

# 2. Combine categories based on rules.

if not is_individual_sponsor:

entity_categories.add(lead_sponsor_category)

entity_categories.update(collaborator_categories)

# If 'Other' exists with more specific categories, it's not a determining factor.

if len(entity_categories) > 1:

entity_categories.discard('Other')

# 3. Apply final categorization logic.

final_category = ''

if not entity_categories:

final_category = 'Other' # e.g., an individual sponsor with no collaborators.

elif len(entity_categories) == 1:

final_category = entity_categories.pop() # Rules 1, 2, 3 & Edge Cases 1, 2

else:

final_category = 'Mixed source' # Rule 4

df.at[index, 'Funding Category'] = final_category

except Exception as e:

df.at[index, 'Funding Category'] = 'Manual Review'

print(f"Error processing row {index} ({lead_sponsor}): {e}")

pbar.update(1)

if (index + 1) % self.progress_save_interval == 0:

self.save_progress(df, temp_file_path)

self.save_final_results(df)

if os.path.exists(temp_file_path):

os.remove(temp_file_path)

except Exception as e:

messagebox.showerror("Error", f"An error occurred during processing: {e}")

self.status_label.config(text="Status: Error during processing.")

def parse_collaborators(self, collaborators_str: str) -> list[str]:

"""

Parses the collaborator string into a list of clean entity names.

This function now removes any parenthetical text (like class tags)

from anywhere within the name.

"""

if not collaborators_str or pd.isna(collaborators_str):

return []

# Split by semicolon to handle multiple collaborators

collaborators = [c.strip() for c in collaborators_str.split(';')]

# --- REFINEMENT ---

# The regex r'\s*\([^)]*\)' finds any text in parentheses and any

# preceding whitespace and removes it. This cleans tags like "(INDUSTRY)",

# "(OTHER)", or "(UNKNOWN)" from anywhere in the string.

cleaned_collaborators = [re.sub(r'\s*\([^)]*\)', '', c).strip() for c in collaborators]

# Return a list of non-empty collaborator names

return [c for c in cleaned_collaborators if c]

def get_entity_category(self, entity_name: str) -> str:

"""

Classifies a single entity name using a "searchless" two-pass verification system.

If the first pass is ambiguous, it makes a second, more forceful API call

to the AI model to re-evaluate the name.

"""

if entity_name in self.classification_cache:

return self.classification_cache[entity_name]

model_name = "gpt-4.1-mini"

valid_categories = {"Industry", "NIH/US Federal", "University/Hospital", "Government", "Research institute/non-profit organization", "Other"}

# --- PASS 1: INITIAL CLASSIFICATION ATTEMPT ---

pass1_system_prompt = (

"You are a classification expert. Analyze the organization's name to determine its primary identity. "

"Respond with only ONE of the following exact category names: 'Industry', 'NIH/US Federal', "

"'University/Hospital', 'Government', 'Research institute/non-profit organization', 'Other'."

)

try:

response = self.client.chat.completions.create(

model=model_name,

messages=[

{"role": "system", "content": pass1_system_prompt},

{"role": "user", "content": f"Organization Name: \"{entity_name}\""}

],

temperature=0.0,

)

category = response.choices[0].message.content.strip().replace("\"", "").replace("*", "")

if category not in valid_categories:

category = 'Other' # Default to Other if the response is invalid

except Exception as e:

print(f"API call failed during Pass 1 for '{entity_name}': {e}")

return "Manual Review"

# --- PASS 2: "SEARCHLESS" CORRECTION ATTEMPT (IF NEEDED) ---

if category == 'Other':

# Do not run a second pass for what looks like a person's name

if len(entity_name.split()) > 4 or any(kw in entity_name.lower() for kw in ['foundation', 'university', 'hospital', 'center', 'gmbh', 'inc', 'stiftung', 'klinikum']):

print(f"Initial classification for '{entity_name}' was 'Other'. Attempting a focused re-evaluation...")

try:

# Step 1: Create a new, more forceful prompt

pass2_system_prompt = (

"You are a verification expert. A previous attempt to classify the following organization resulted "

"in 'Other', which is likely incorrect. You must re-evaluate the name. Analyze it for linguistic "

"clues (e.g., 'Foundation', 'Stiftung', 'Klinikum', 'University', 'Hospital', 'GmbH', 'AG') and its "

"likely function. You must choose one of the specific categories. Do not respond with 'Other' "

"unless it is 100% certain to be an individual person's name.\n\n"

"**Specific Categories:**\n"

"- Industry\n- NIH/US Federal\n- University/Hospital\n- Government\n- Research institute/non-profit organization"

)

# Step 2: Make the second, more forceful API call

final_response = self.client.chat.completions.create(

model=model_name,

messages=[

{"role": "system", "content": pass2_system_prompt},

{"role": "user", "content": f"Re-evaluate this name: \"{entity_name}\""}

],

temperature=0.0

)

verified_category = final_response.choices[0].message.content.strip().replace("\"", "").replace("*", "")

specific_categories = {"Industry", "NIH/US Federal", "University/Hospital", "Government", "Research institute/non-profit organization"}

if verified_category in specific_categories:

print(f"Verification successful. Corrected category for '{entity_name}' is '{verified_category}'.")

category = verified_category

except Exception as e:

print(f"Error during re-evaluation (Pass 2) for '{entity_name}': {e}")

# Fallback to the original 'Other' category if the second pass fails

self.classification_cache[entity_name] = category

return category

def save_progress(self, df, temp_file_path):

"""Saves the current progress to a temporary file."""

try:

df.to_excel(temp_file_path, index=False)

self.status_label.config(text=f"Status: Progress for {os.path.basename(temp_file_path)} saved.")

self.root.update_idletasks()

except Exception as e:

print(f"Failed to save progress: {e}")

def save_final_results(self, df):

"""Saves the final results to a new Excel file."""

dir_name, file_name = os.path.split(self.file_path)

base_name, ext = os.path.splitext(file_name)

self.output_file_path = os.path.join(dir_name, f"{base_name}_categorized_final{ext}")

df.to_excel(self.output_file_path, index=False)

self.status_label.config(text="Status: Processing complete!")

self.output_label.config(text=f"Categorized data saved to:\n{self.output_file_path}")

messagebox.showinfo("Processing Complete", f"Categorization finished!\nResults saved to: {self.output_file_path}")

# Main execution

if __name__ == "__main__":

root = tk.Tk()

app = SponsorCategorizerApp(root)

root.mainloop()
